# Editorial: Insights in the emergence and persistence of COVID-19: a modelling perspective

**DOI:** 10.3389/fepid.2024.1504677

**Published:** 2024-10-29

**Authors:** Carl J. E. Suster, Sheryl L. Chang

**Affiliations:** ^1^ Centre for Infectious Diseases and Microbiology - Public Health, Westmead Hospital, Westmead, NSW, Australia; ^2^ Sydney Infectious Diseases Institute, Faculty of Medicine and Health, The University of Sydney, Westmead, NSW, Australia; ^3^ Centre for Complex Systems, Faculty of Engineering, The University of Sydney, Camperdown, NSW, Australia

**Keywords:** COVID-19 modelling, epidemic models, public health interventions, mobility data, population heterogeneity

**Editorial on the Research Topic**
Insights in the emergence and persistence of COVID-19: a modelling perspective

As the acute phase of the COVID-19 pandemic developed, jurisdictions needed to act quickly to implement public health interventions to manage the emerging threat. Border closures, restrictions on gatherings and mobility, and vaccination campaigns were rolled out with at times unprecedented scale and velocity. Mathematical modelling was an important tool in providing evidence to inform these responses. A plethora of new models and modelling approaches were developed to make use of available data and to address critical questions of public health responders and decision makers.

The world has now moved beyond the acute phase of the pandemic and into a phase of persistent circulation of the SARS-CoV-2 virus. We must reflect on our collective experience with modelling thus far. Did modelling do as well as it could have in the early days of the pandemic? Are the modelling tools we have now adequate for the new set of challenges in managing the persistent phase? Will they meet our needs when the next pandemic arrives? This research topic brings together four articles that explore these questions.

One of the most pressing tasks required of modelling is to forecast the incidence of new cases. Accurate forecasts can facilitate efficient mobilisation of healthcare resources and balancing the cost of potential interventions (both in economic terms and otherwise) against the expected burden of disease and rate of transmission. Sudhakar et al. retrospectively evaluate forecasts of a modelling consortium in the United States. They use this analysis to examine limitations of compartmental models in general, finding evidence of structural bias in the approach. They argue that compartmental models are predicated on unrealistic assumptions about how populations move and interact.

One way to address these limitations is to incorporate realistic mobility data. The Google COVID-19 Aggregated Mobility Research Dataset (GAMRD) contains aggregate information about journeys by users of the company's smartphone software. Berman et al. present their model combining a compartmental model with a contact network calibrated with the GAMRD dataset. They demonstrate the model's utility in predicting vulnerable towns and high-risk population flows in the sparsely populated state of Western Australia.

Mobility at the level of towns is at a proxy for data about real contacts between individuals, which is normally collected retrospectively (if at all) as part of follow-up after diagnosis of a case. During the pandemic, some governments and their partners developed smartphone apps that could prospectively record contacts between people and share anonymised data with authorities after a case was identified. Fosch et al. present a model to investigate why evaluations of these digital contact tracing apps have reported mixed conclusions on their effectiveness. They provide recommendations to policymakers should the technology be called upon again in future.

Vaccination and lockdowns were key interventions adopted by many jurisdictions in the pandemic. Once vaccines arrived, jurisdictions were faced with decisions about how to deploy limited stocks to best protect the population and allow mobility restrictions to be lifted. Bonsall et al. model this as an optimisation problem with the objective of minimising mortality. They find that vaccination strategies which prioritise vulnerable populations can result in inefficiencies due to the resulting inhomogeneous distribution of vaccinated individuals.

The importance of realistic models of population distribution and mixing is a recurring theme in the articles of this research topic. They suggest that there remains much work for disease modellers. We need models that can respond to changing public health needs, can incorporate new data streams and technologies, and avoid treating populations as homogenous aggregates.

